# Architectural modelling for robotics: RoboArch and the CorteX example

**DOI:** 10.3389/frobt.2022.991637

**Published:** 2022-10-28

**Authors:** Will Barnett, Ana Cavalcanti, Alvaro Miyazawa

**Affiliations:** Department of Computer Science, University of York, York, United Kingdom

**Keywords:** software engineering, patterns, RoboStar framework, nuclear industry, verification, simulation, test, proof

## Abstract

The need for robotic systems to be verified grows as robots are increasingly used in complex applications with safety implications. Model-driven engineering and domain-specific languages (DSLs) have proven useful in the development of complex systems. RoboChart is a DSL for modelling robot software controllers using state machines and a simple component model. It is distinctive in that it has a formal semantics and support for automated verification. Our work enriches RoboChart with support for modelling architectures and architectural patterns used in the robotics domain. Support is in the shape of an additional DSL, RoboArch, whose primitive concepts encapsulate the notion of a layered architecture and architectural patterns for use in the design of the layers that are only informally described in the literature. A RoboArch model can be used to generate automatically a sketch of a RoboChart model, and the rules for automatic generation define a semantics for RoboArch. Additional patterns can be formalised by extending RoboArch. In this paper, we present RoboArch, and give a perspective of how it can be used in conjunction with CorteX, a software framework developed for the nuclear industry.

## 1 Introduction

Robotic systems are being used in an increasingly diverse range of applications, and in more dynamic and unstructured environments. With autonomy and the ability to operate in close proximity to humans, safety becomes an issue. Furthermore, robotic systems and their software are becoming more complex. In previous work, we have contributed to the verification of robotic systems using a domain-specific language with a formal semantics, namely, RoboChart (Miyazawa et al., 2017, 2019).

In this paper, we present an approach to defining RoboChart models for software that use architectures of wide interest in robotics. It is based on a novel domain-specific notation, RoboArch, presented here for the first time. It embeds robotics software architectural concepts and enables automatic generation, *via* model transformation, of partial RoboChart models, that is, sketches of RoboChart models that can be completed by designers with application-specific descriptions (of actions and state machines).

The definition of a system’s architecture during its design has been considered a beneficial technique as the scale of software systems has grown. The architecture provides a structural representation that enables the evaluation of system attributes and of alternative system designs and modifications (Bass et al., 2012). From experience, practitioners have identified structures and relationships within system architectures that solve recurring problems. These solutions have been generalised as architectural patterns that are reusable in the design of new systems (Gamma et al., 1995). For the robotics domain, some common patterns have emerged: notably, the use of layers for robot control (Siciliano and Khatib, 2016, pp. 286–289).

In many other complex multidisciplinary domains, Model-Driven Engineering (MDE) is being used successfully to mitigate complexity (Franz et al., 2018). The core principle of MDE is to use abstract models of a system as the primary artefact(s) of its development process. This promotes identification of the underlying concepts free from specific implementation dependencies. The use of abstract models also facilitates the automation of the software development process. In this way developers can devote their time to understanding and solving the domain-specific problems.

Domain-specific languages (DSL) facilitate the development of models by embedding core concepts of a target domain, and enabling the definition of concise representations understood by practitioners. This avoids the need for each development team to identify these concepts, resulting in duplication of work and hindering reusability. Over the last 25 years, there have been considerable developments in MDE for robotics, with the creation of many DSL for its different sub-domains (Nordmann et al., 2016).

Some examples of DSL for robotics include: RobotML (Dhouib et al., 2012), SmartSoft (Stamper et al., 2016), and BCM (Bruyninckx et al., 2013). These DSLs, like the majority available, do not have formally defined semantics (Cavalcanti et al., 2021b). Therefore, the support for formal verification of robotic systems is limited. A recent literature survey (Luckcuck et al., 2019) found sixty-three examples of the application of formal methods within the robotics domain. Formal methods enable the early verification (proof, simulation, and testing) of a system through the use of rigorous automated techniques with mathematical foundations. Early use of verification techniques and high levels of automation enable the development of systems that are more reliable and cheaper.

RoboChart is a DSL for modelling robotics software controllers using state machines and a simple component model; RoboChart makes innovative use of formal methods for automated verification. The associated tool, RoboTool^1^, provides features of MDE, which include a graphical interface for creating models, and automatic generation of source code and mathematical descriptions. Additionally, RoboChart supports automatic verification of properties such as deadlock and livelock freedom using model checking, along with semi-automatic verification techniques using theorem proving (Cavalcanti A. L. C. et al., 2021).

To date, RoboChart has been used to model more than twenty proof-of-concept case studies. They have facilitated the development and demonstration of RoboChart and its verification technology. None of them, however, adopt an elaborate software architecture. For larger robotic systems, support for modelling taking advantage of commonly used architectural patterns can enable explicit modelling of the structure of systems with potential to assist in reuse and compositional design and reasoning.

RoboArch allows the description of layered designs for robotic control software, and of design patterns for each layer. In this paper, we not only give an overview of RoboArch *via* a motivating example, but also present its complete metamodel and set of well-formedness conditions that specify the valid RoboArch models. We also describe our model-transformation approach, based on 50 rules, mechanised to generate automatically a sketch of a RoboChart model from a RoboArch architectural design of a system.

Besides supporting the description of architectural designs, RoboArch formalises a notion of a layered architecture and other patterns. Most of these patterns are described in the literature only informally, sometimes with different variations described by different authors. At best, patterns are realised in an implementation or programming language. Such descriptions necessarily mix the core concepts of the architectural patterns with those of the application or programming language. In contrast, the RoboArch formalisation identifies the core concepts of a pattern and their relationship.

The CorteX framework (Caliskanelli et al., 2021) has been designed for use in nuclear robotics to address the challenges of developing their complex robotic systems that need to be maintained over long periods of time, often to deal with changing requirements due to the unknown operational conditions. CorteX favours the development of maintainable and extensible systems through specialised data and communications designs. Designs for the CorteX middleware are inherently concurrent.

Our vision is the alliance of RoboArch and CorteX to support 1) the identification and formalisation of the architectural designs that rely on CorteX and 2) the elicitation of assurance evidence to increase confidence in CorteX-based software and support the construction of assurance arguments. By integrating CorteX with RoboArch, and, *via* RoboArch, to RoboChart, we connect CorteX to the RoboStar approach to Software Engineering for Robotics (Cavalcanti A. L. C. et al., 2021). With that, we enable, automatic generation of mathematical models that specify the meaning of the RoboArch designs, and automatic and semi-automatic verification of properties *via* model-checking and theorem proving. Further specialisation of the approach can lead to automatic generation of CorteX code for simulation and deployment.

Our novel contributions in this paper are as follows.

1) Design of RoboArch for description of layered architectures for robotic control software.
2) Definition of the metamodel and well-formedness conditions of RoboArch.
3) Description of a technique for model-to-model transformation from RoboArch to RoboChart.
4) Formalisation of the reactive-skills architectural pattern for design of control layers, illustrating a general approach to formalise patterns using RoboArch and RoboChart.
5) Discussion of perspectives for allying the use of RoboArch and CorteX, and, in particular, of the formalisation of CorteX in RoboArch and RoboChart.

These results enable use of MDE in the development of control software for robotic systems in a way that focusses on use of well-known patterns allied with the advantages of modern verification techniques. RoboChart, and, therefore, RoboArch, are part of a design and verification framework, called RoboStar, that supports automated generation of simulations, tests, and proof.

In the next section, we describe related work on architectures for robotics. Section 3 presents RoboArch: metamodel, well-formedness conditions, and translation to RoboChart. Section 4 shows how a design pattern can be formalised in RoboArch using the example of reactive skills. In Section 5 we conclude, discussing our approach for the integration of RoboArch and CorteX as future work.

## 2 Related work

In this section, we discuss the literature on architectural patterns for robotics. Crucially, this justifies our choice of layer as a core concept in RoboArch, but also indicates other patterns of interest, including reactive skills, which we also formalise in this paper.

RoboArch is not related to the homonym in (Bonato and Marques, 2009), which is a tool to support the development of mobile robots. The focus in (Bonato and Marques, 2009) is on implementation, not modelling, of hardware-software co-designs based on hardware and software components, and code generation for FPGA, not software architectures. Moreover, there is no semantics or support for verification beyond simulation for the notation adopted by RoboArch to define the compositions.

Other works that share our aim to reduce effort in the development of control software in robotics focus on the programming, rather than the modelling, level. The result is a variety of middleware, encouraging code reuse and component-based development (Bruyninckx, 2001; Metta et al., 2006; Ando et al., 2008; Chitta et al., 2017; Muratore et al., 2017). These works provide useful resources for programming, but do not address the issues arising from a code, rather than model-based, approach to development. Work on RoboArch and RoboChart is complementary. In particular, we consider here how we can provide direct support for use of the modern CorteX middleware that has a track record in the nuclear industry.

Historical architectural patterns include Sense Plan Act (SPA) (Siciliano and Khatib, 2016, *p*. 285) and subsumption (Brooks, 1986). SPA is an example of a pattern that is deliberative: time is taken to plan what to do next, and then the plan is acted out with no sensing or feedback during acting. A robot using SPA in a dynamically changing world can be slow and error prone in response to environmental change.

Conversely, subsumption is an example of an architectural pattern that is reactive, where the environment is constantly sensed and used to directly shape the robot’s actions. A robot using subsumption responds rapidly to a changing world; however, complex actions are difficult to achieve.

More recent hybrid architectural patterns combine the principles from SPA and subsumption to benefit from both the deliberative and reactive properties. In total, twenty-two architectural patterns used by robotics systems have been identified from the literature; these are listed in Table 1. Five have been selected for discussion based upon evidence of application, reuse, and activity of development. The collective publications that focus on an architectural pattern have been used to find evidence of application, with the scale of any documented application used to give preference to patterns that have been used in large deployments in the real world. The number of publications where an architectural pattern was used in a new application has been used to asses reuse. Finally, preference has been given to patterns with recent activity, determined by the date and frequency of publications where the pattern has been used.

**TABLE 1:** The patterns identified from the literature.

Pattern	Focus	Year
CoSiMA Wigand et al. (2018)	Safe real-time robots	2018
^a^IRSA Backes et al. (2018)	Autonomous robots	2018
^a^SERA García et al. (2018)	Decentralised teams	2018
Aerostack Sanchez-Lopez et al. (2017)	Autonomous unmanned aerial systems	2017
^a^CARACaS Huntsberger and Woodward, (2011)	Autonomous robots	2011
EFTCoR Álvarez et al. (2006)	Service robot control	2006
Syndicate Sellner et al. (2006)	Autonomous teams	2006
DDX (Corke et al., 2004)	Distributed robot controllers	2004
^a^CLARAty (Volpe et al., 2001)	Autonomous robots	2001
HARPIC (Luzeaux and Dalgalarrondo, 2001)	Autonomous robots	2001
^a^LAAS (Alami et al., 1998)	Autonomous robots	1998
Remote Agent (Muscettola et al., 1998)	Autonomous robots	1998
ORCCAD (Borrelly et al., 1998)	Robot control	1998
Planner Reactor (Lyons and Hendriks, 1995)	Autonomous robots	1995
Reactive Skills (Yu et al., 1994)	Autonomous robots	1994
CIRCA (Musliner et al., 1993)	Real-time intelligent robots	1993
ATLANTIS (Gat, 1992)	Autonomous robots	1992
Layered Competencies (Bonasso, 1991)	Autonomous robots	1991
Motor Schema (Arkin, 1989)	Robot control	1989
NASREM (Albus et al., 1989)	Autonomous robots	1989
AuRA (Arkin, 1987)	Autonomous robots	1987
Subsumption (Brooks, 1986)	Autonomous robots	1986

^a^ Selected for further discussion.

**LAAS** was developed at LAAS^2^ in 1998 for autonomous robots. A fundamental goal of LAAS is to provide both deliberative and reactive capabilities required for autonomy.

The LAAS pattern is made up of the following three layers. The *Functional Layer* provides basic robot actions that are organised into modules consisting of processing functions, task loops, and monitoring functions for reactive behaviour. An *Execution Control Layer* selects functions from the functional layer to carry out sequences of actions determined by the decision layer. Finally, the *Decision Layer* plans the sequence of actions necessary to achieve mission goals and supervises the execution of the plans.

The functional layer consists of a network of modules that provide services related to a particular sensor, actuator, or data resource of the robot. All modules have a fixed generic structure made up of a controller and execution engine. A tool can be used to generate module source code. The services provided by the modules are accessed by the executive layer above and other modules from the functional layer through the use of a non-blocking client-server communication model.

The execution control layer bridges the slow, high-level, processing of the decision layer, and the fast, low-level, control of the functional layer. It has an executive module that takes sequences of actions from the decision layer, and selects and triggers the functions that the functional layer must carry out. In addition, the executive receives replies from the functional layer and reports activity progress to the decision layer.

The decision layer has one or more pairs of a supervisor and a planner. The supervisor takes a sequence of actions from the planner and manages their execution by communicating them to the execution layer, and responding to reports received from it. The planner creates a sequence of actions to achieve a goal. The supervisor also passes down situations to monitor and associated responses within the constraints of the plan. These responses enable the lower layers to react without the need for involvement of the decision layer, improving response time and reducing unnecessary replanning.

LAAS has been used in the implementation of the ADAM rough terrain planetary exploration rover (Chatila et al., 1995), and of three Hilare autonomous environment exploration robots as part of the MARTHA European project. More recently, Behaviour Interaction Priority (BIP) models have been used to verify the functional layer of the LAAS pattern (Silva et al., 2015).

**CLARAty** (Coupled Layer Architecture for Robotic Autonomy) was developed at NASA in 2001 for planetary surface-exploration rovers. CLARAty is designed to be reusable and to support multiple robot platforms; it consists of two-layers: a functional layer, and a decision layer formed by combining the planning and executive layers from a three-layer architecture. A key concept defined in CLARAty is granularity, which reflects the varying levels of deliberativeness available to the robotic system.

The functional layer provides a software interface to the hardware capabilities of the robot, and it is structured using an object-oriented hierarchy. At the top of the hierarchy is the Robot superclass from which everything inherits. At subsequent levels down the hierarchy, classes are less abstract and each provide functionality for a piece of the robot’s hardware. At the bottom of the hierarchy, each class provides access to a specific piece of hardware functionality and its current state.

Classes can provide functionality that requires minimal input from the decision layer, therefore, this type of class can be considered more reactive. For example, the class for a rover may offer a method for obstacle avoidance. Alternatively classes can provide functionality that requires regular input from the decision layer, therefore, the class can be considered more deliberative. For example, the class for a robotic arm may offer a method for setting the position for one of its five motors.

The single decision layer enables state information between planner and executive to be shared, which means that the planner becomes tightly integrated with the executive. Consequently, discrepancy between the planner and the functional layer’s state is minimised.

The CLARAty pattern has been used for a variety of robot platforms: Rocky 8, FIDO, ROCKY 7, K9 Rovers, and ATRV Jr COTS platform (Nesnas et al., 2006). The different platforms have a variety of deployment architectures, from a single processor requiring hard real-time scheduling, to distributed microprocessors using soft real-time scheduling.

**CARACaS** (Control Architecture for Robotic Agent Command and Sensing) is an architectural pattern developed at NASA in 2011 for control of autonomous underwater vehicles (AUV), and autonomous surface vehicles (ASV). CARACaS-based software supports operation in uncontrolled environments ensuring the vehicles obey maritime regulations. A CARACaS design supports cooperation between different vehicles and makes use of dynamic planning to adapt to the current environmental conditions and mission goals.

The five main elements of CARACaS are as follows. *Actuators* interface the actuators of the vehicle. A *Behaviour Engine* coordinates and enables the composition of behaviours acting on the actuators. The arbitration mechanisms controlling the enabling and disabling of behaviours are subsumption, voting, and interval programming. A *Perception Engine* creates maps for safe navigation and hazard perception from the sensors. A *Dynamic Planning Engine* chooses activities to accomplish mission goals while observing resource constraints. For that, it uses Continuous Activity Scheduling Planning Execution and Replanning (CASPER) (Chien et al., 2000), and issues commands to the Behaviour Engine. Finally, a *World Model* contains state information including plans, maps, and other agents.

Layers are not defined in Huntsberger and Woodward (2011), but a CARACaS design can be partitioned into two layers. At the lowest level, a behavioural layer includes the Actuators, and the Behaviour and Perception Engine elements. The higher layer consists of the Dynamic Planning and the World Model.

Although CARACaS is targeted at autonomous water-based vehicles, it contains all of the required elements to be applied more generally as a pattern for the control of robots.

**IRSA** (Intelligent Robotics System Architecture) was developed at NASA in 2018 to streamline the transition of robotic algorithms from development onto flight systems by improving compatibility with existing flight software architectures. IRSA uses concepts from other patterns: CARACaS and CLARAty.

The main elements of IRSA are as follows. A *Primitive* provides low-level behaviours that can have control loops. *Behaviour* provides autonomy, transitioning between multiple states during execution. The *Executive* receives and executes a sequence of instruction commands from the planner. The *Planner* uses the system state from the world model to produce the sequence of command instructions. A *Sequence* contains the instructions that the robot must perform. A *Verifier* verifies whether the sequence is valid. Finally, the *Robot World Model* maintains a model of the robot with local and global state information.

An IRSA design can be mapped onto a three-layer pattern with a common world model accessible to all layers. The behavior and the primitive elements provide control over the robot; so, these two elements can be placed in the bottom layer. The executive receives sequences of commands and manages their execution using the behaviours. Therefore, the executive is the middle layer. The planner uses the state of the system from the world model to create a sequence of commands checked by the verifier. Therefore, the planner, sequence, and verifier elements are in the layer above the executive.

The IRSA architectural pattern has been deployed on a variety of test beds: comet surface sample return, Europa lander, Mars 2020 Controls and Autonomy, and the RoboSimian DARPA challenge.

**SERA** (The Self-adaptive dEcentralised Robotic Architecture) has been developed at the Chalmers University of Technology in 2018. SERA’s primary goal is to support decentralised self-adaptive collaboration between robots or humans, and it is based on the three-layer self-management architectural pattern. SERA has been evaluated in collaboration with industrial partners in the Co4Robots H2020 EU project.

The layers of the SERA pattern are as follows. The *Component Control Layer* provides software interfaces to the robot’s sensors and actuators, grouped into control action components responsible for particular areas of functionality. The *Change Management Layer* receives the local mission and creates a plan in order satisfy its goals. It executes the plan by calling appropriate control actions from the component control layer. Finally, the *Mission Management Layer* manages the local mission for each robot and communicates with other robots in order to synchronise and achieve the global mission.

The mission management layer receives a mission specification from a central station as a temporal logic formulae. The mission manager checks its feasibility and, if it is feasible, passes the mission to the adaptation manager in the layer below. If the mission is infeasible, a communication and collaboration manager communicates and synchronises with the other robots involved in the mission. During the synchronisation, an updated achievable mission that meets the original mission specification is computed.

This pattern places more functionality in the lowest component control layer. A key feature of SERA is communication among robots, which provides greater flexibility in achieving the mission goals.

### 2.1 Discussion

Generally no particular pattern or selection of patterns are widely used. There is a tendency for each project to establish its own pattern. Between research groups, however, there is some reuse of patterns.

Layers are a common theme among many of the recent architectural patterns. Even when layers have not been explicitly specified, the elements of a pattern are structured such that they can be mapped onto a layered architectural pattern. All patterns have a functional layer that interacts with the robots sensors and actuators. The upper layers following the functional layer vary in number and purpose.

The functional layer is required by all architectural patterns because every robot requires a means to sense and interact with its environment. From the patterns surveyed, this layer can be categorised as either service or behavioural. CLARAty, LAAS and SERA are all examples of patterns that have a service-based functional layer, whereas, CARACaS and IRSA have behavioural-based functional layers.

Examples of behavioural control patterns that can be used for functional layer include subsumption (Brooks, 1986) and reactive skills as used by the control layer of 3T (Bonasso et al., 1997). It is common for the decision layer to be placed directly above the functional layer.

Patterns that do not use an executive layer take different approaches to managing the system’s state. For instance, SERA and CLARAty use information in the decision layer to hold system state. Whereas, CARACaS uses a world model layer that is accessible by all other layers to hold system state.

Some patterns such as SERA have an additional social layer for collaboration between teams of robots. Similarly LAAS supports this through adding supervisor-planner pairs, but considers this to be an extension of the decision layer rather than a new layer. Generally the layered pattern lends itself to the addition of new layers for extending the level of system capability.

RoboArch directly supports the definition of layered architectures, with an arbitrary number of layers. A degenerate layered architecture with just one layer can be used to define a design that does not actually uses layers. As indicated above, however, the use of more elaborate layers, some using specific patterns themselves, is common. In what follows, we present the RoboArch notation.

## 3 Materials and methods: RoboArch

In this section, we show how a layered design can be described using RoboArch. We give an overview using the example of an office delivery robot from (Siciliano and Khatib, 2016, pp. 291–295) (Section 3.1). In Section 3.2 we present the complete metamodel and well-formedness conditions of RoboArch. Finally, in Section 3.3, we describe the RoboChart model defined by a RoboArch design. In the next Section 4, we show an example of how a pattern for the control layer can be characterised and used.

### 3.1 Overview

**FIGURE 1:**
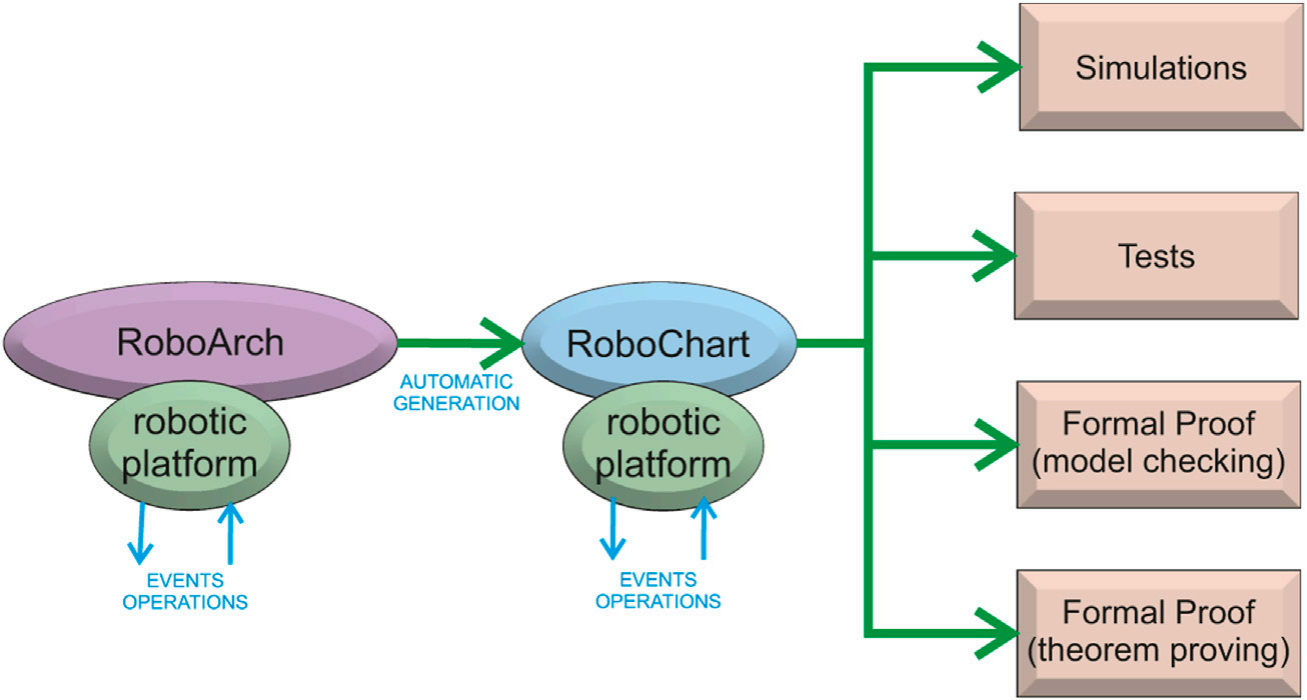
RoboArch in the context of RoboStar. With a RoboArch architectural design, we can generate automatically a sketch of a RoboChart behavioural model. Using the RoboChart model, we can take advantage of a plethora of modern verification techniques supported by automated generation of artefacts.

RoboArch is a self-contained notation that can be used independently. As mentioned, however, its semantics is given by translation rules that define a (sketch of a) RoboChart model. This not only gives RoboArch a precise and formal semantics, but also paves the way for the use of the RoboStar framework to design and verify the control software. Figure 1 gives an overview of the possibilities.

As indicated in Figure 1, a key concept in RoboArch is that of a robotic platform. RoboArch designs are platform independent, so the robotic platform here describes the services the robot provides that can be used in the development of the control software. The services are abstractions of the robot’s sensors and actuators defined *via* the declaration of input and output events and operations that can be realised *via* actual sensors and actuators. The same approach is taken in RoboChart.

To give an overview of the RoboArch notation, we consider the example of a robot whose goal is to deliver items of post within a typical office building, transporting them from a central mailroom to each of the offices within the building. To achieve its goal the robot must safely navigate along the corridors of the building while avoiding any obstacles such as people and furniture.

Listing 1 A system and its type declarations.

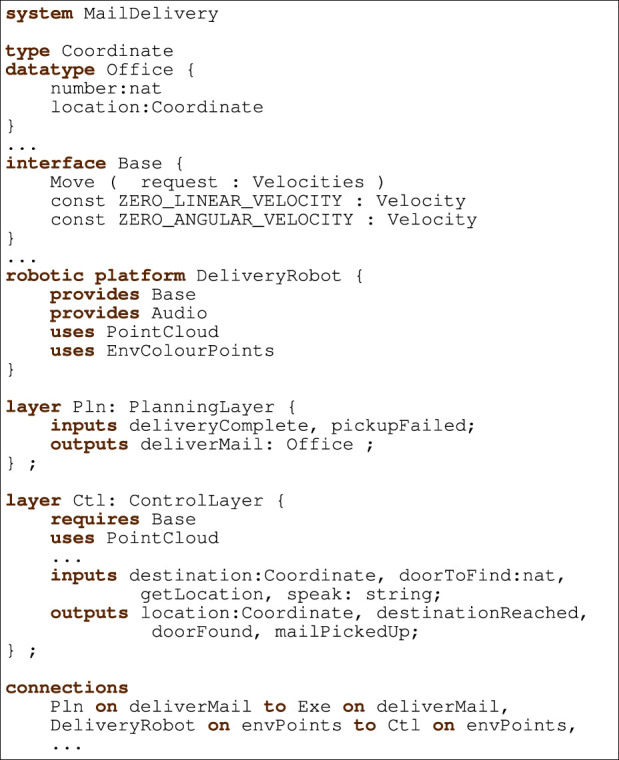

A RoboArch model for the mail delivery system is sketched in Listing 1. A system clause gives a name to a model and introduces the outer scope to define the layers and the robotic platform. The robotic platform must be used by a single layer, usually the control layer. In addition to the architectural elements, a RoboArch model also contains definitions for types, functions, interfaces, and connections. For our example, Line 1 of Listing 1 declares the system with the name MailDelivery.

RoboArch adopts the type constructors and typing rules of the well-established data modelling notation Z (Woodcock and Davies, 1996), allowing the definition of primitive types, records, sets, and so on. RoboChart and all RoboStar notations adopt the same typing approach. By adopting the Z type system, we benefit from a well-known powerful type system, which has the expected facilities to define a rich, possibly abstract, data model, and that is supported by verification tools. In our example, the next few lines define types. Most type definitions are omitted here, but the complete example is available^3^.

Robotic platforms are normally defined in terms of interfaces. For our example, the robotic platform is named DeliveryRobot, and its definition references interfaces Base, Audio, PointCloud, and EnvColourPoints, some omitted in Listing 1. Interfaces group events or operations, and are referenced using provides and uses clauses in a platform definition. The Base interface models the interactions that control movement. There is one operation Move and two constants. Move is an abstraction for motor functionality that can be accessed by the software *via* a call to this operation. It is a service provided by the platform, since Base is declared in a provides clause. The interfaces declared in uses clauses contain events that represent points of interaction (inputs and outputs), corresponding to inputs from sensors, or outputs to actuators. They are used by connecting the platform events to those of a layer.

The design in Listing 1 is a typical three-layer architecture. Every layer has a unique name, and optionally can have a type, a pattern, inputs and outputs. The three specific layer types are ControlLayer, ExecutiveLayer, and PlanningLayer. We can also not provide a type so that a customised architectural structure can be defined. The services of a layer are accessed through its inputs and outputs.

The layer clause is used to define the layer name and type. In Listing 1, we show a layer with name Pln and type PlanningLayer. It has one output deliverMail of type Office that requests the number of the office to which mail is currently being delivered. There are two inputs deliveryComplete and pickupFailed that have no associated value type; their occurrence indicates an outcome of the currently requested delivery. The inputs and outputs are used to communicate with another layer or the robotic platform; in our example communication is with an executive layer, omitted in Listing 1.

A layer of control type can directly communicate with a robotic platform, and so reference platform interfaces. The control layer for our example is Ctl. Its inputs and outputs communicate with the executive layer and DeliveryRobot. The requires and uses clauses reference the interfaces with the operations of the platform that it requires and, the events that it uses. While an ExecutiveLayer and a PlanningLayer cannot require or use services of a platform, a generic layer also can.

The connections among the layers and the robotic platform are defined under a system’s connections clause. Each connection is unidirectional and connects an input or output on a layer or event of the platform to another. Listing 1 shows some of the connections for our mail delivery example. For example, the first declares a connection from the Pln layer’s deliverMail output event to an Exe layer’s deliverMail input event. The second connection is between the robotic platform (DeliveryRobot) and the control layer (Ctl). Several other connections are omitted.

In the next section, we give a complete description of the structure of RoboArch designs.

### 3.2 Metamodel and well-formedness

**FIGURE 2:**
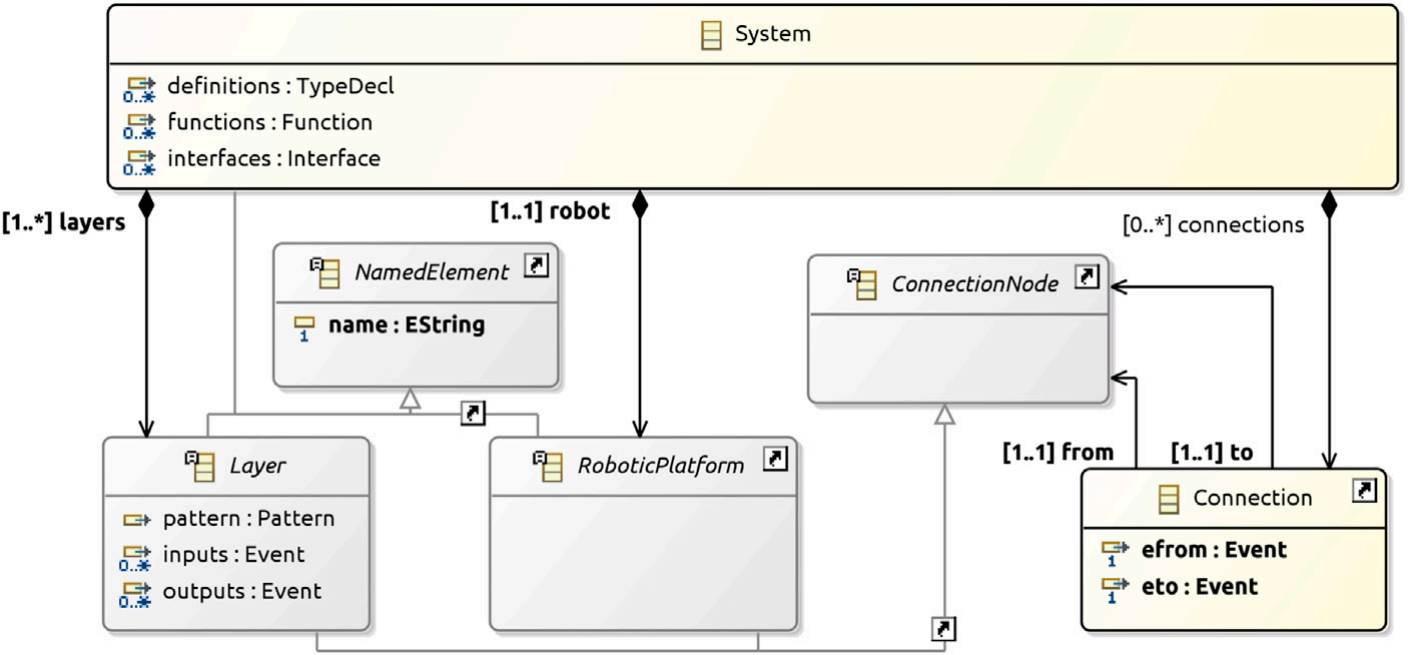
System metamodel.

Figure 2 presents the RoboArch metamodel: the classes, and their attributes and associations, that represent a RoboArch design. The main class is System, whose objects have definitions of layers, robotic platform, connections, definitions, functions, and interfaces. The classes TypeDecl, Function, and Interface defining types for attributes of System come from the RoboChart metamodel (Miyazawa et al., 2020).

The RoboticPlatform class also comes from the RoboChart metamodel. RoboticPlatforms have a name and can declare events and variables as well as reference interfaces.

Layers can optionally have a pattern that defines their behaviour. (An example is presented in the next section.) Layers can also have inputs and outputs, which are Events, a concept also from RoboChart. An Event can have a type, which, if present, defines the values that can be communicated.

Systems, RoboticPlatforms and Layers are NamedElements: they have a name attribute. RoboticPlatforms and Layers are also ConnectionNodes: elements that can be connected *via* their events. Connections are between a source efrom and a target eto event that belong to the to and from ConnectionNodes.

**FIGURE 3:**
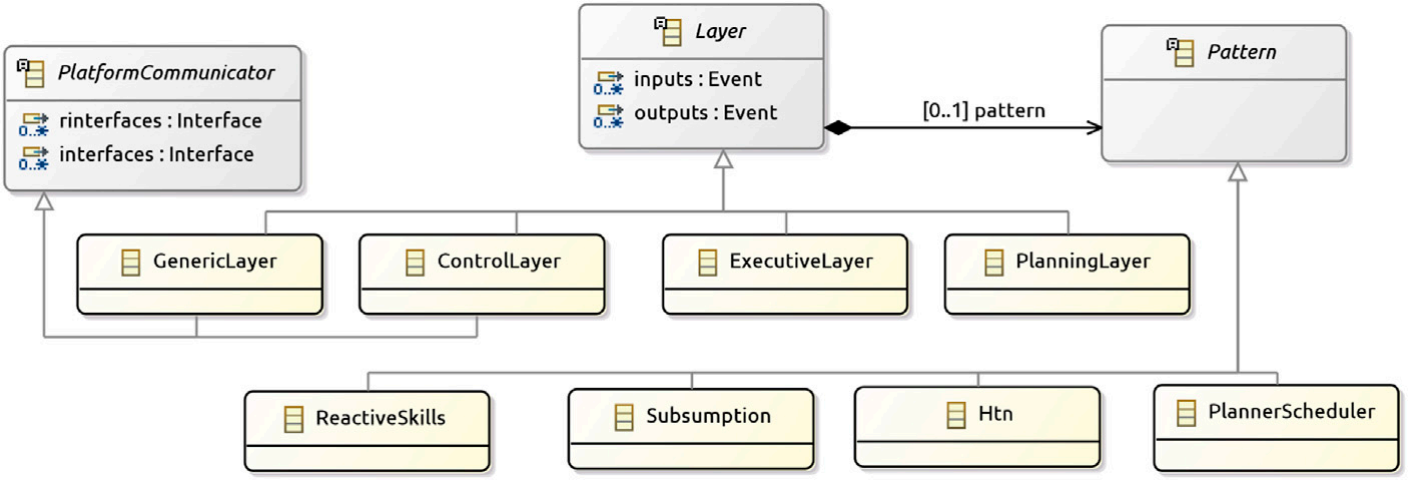
Layers metamodel.

Layer is further defined in Figure 3; it has four subclasses. A GenericLayer represents the most general kind of layer, without a declared type, offering flexibility to model systems with minimal restrictions. The three other kinds of layers, ControlLayer, ExecutiveLayer, and PlanningLayer, have specific well-formedness conditions (discussed later) that characterise the connections and patterns of a valid architectural design.

GenericLayers and ControlLayers can communicate with the RoboticPlatform. They are, therefore, represented by subclasses of an abstract class PlatformCommunicator. The objects of this class have required and defined attributes that record the interface declarations.

As mentioned, Layers can have a pattern. Figure 3 includes examples of patterns represented by subclasses of Pattern, namely, ReactiveSkills, Subsumption, Htn, and PlannerScheduler. To formalise a pattern for use in RoboArch designs, we need to add a subclass of Pattern to represent it. In the next section, we detail ReactiveSkills as an example of how a pattern can be formalised. Section 5 describes a RoboArch pattern for CorteX designs. Current work is considering the formalisation of Subsumption and Htn.

Not all models that can be created obeying the metamodel are valid. For instance, considering just the restrictions defined by the metamodel, we can create an architecture that connects events of different types. No typing rules are captured in the metamodel. As another example, the metamodel allows the specification of an architecture without connections with the robotic platform. Such design is for a software that does not carry out any visible task, and we regard it as invalid. Although we could translate such designs to RoboChart, there is little point in delaying the identification of problems by working with invalid designs.

Instead, we define well-formedness conditions, presented here in Table 2, which characterise the valid designs. These conditions provide modellers with additional guidance and support for validation when defining an architecture.The conditions can be checked by the RoboArch tool.

**TABLE 2:** The well-formedness conditions of RoboArch.

Condition	Description
**S1**	A System must have one or more connections that relate a single Layer to a RoboticPlatform or there must be a Layer that has at least one or more required interfaces (elements in rinterfaces)
**S2**	For Systems with more than one Layer, each Layer must have at least one input or output
**S3**	For Systems with more than two Layers, their ordering given by Connections must be: ControlLayers < ExecutiveLayers < PlanningLayers
**S4**	Connections must associate a Layer with at most two other Layers
**S5**	Connections involving the ControlLayer must associate it with at most one other Layer
**S6**	The connections of a System must associate events defined by interfaces of GenericLayers and ControlLayers with events of the RoboticPlatform
**S7**	Connections efrom and eto event types must match
**S8**	Connections must connect Layer inputs to outputs or *vice versa*

S1 ensures that it is possible to interact with the platform. Because a Layer must provide a service, S2 ensures that it provides a means for external interaction. S3 ensures that, if used, the ExecutiveLayer is the middle intermediate layer between the planning and control layers with no direct communication between them. S4, S5, and S6 are concerned with the proper use of layers, without bypassing communications, and creating inappropriate dependencies. S7 prevents type errors, and S8 ensures correct data flow.

In the next section we describe how a RoboArch design can be formalised in RoboChart, and how transformation rules can be used to generate RoboChart models.

### 3.3 RoboArch in RoboChart

**FIGURE 4:**
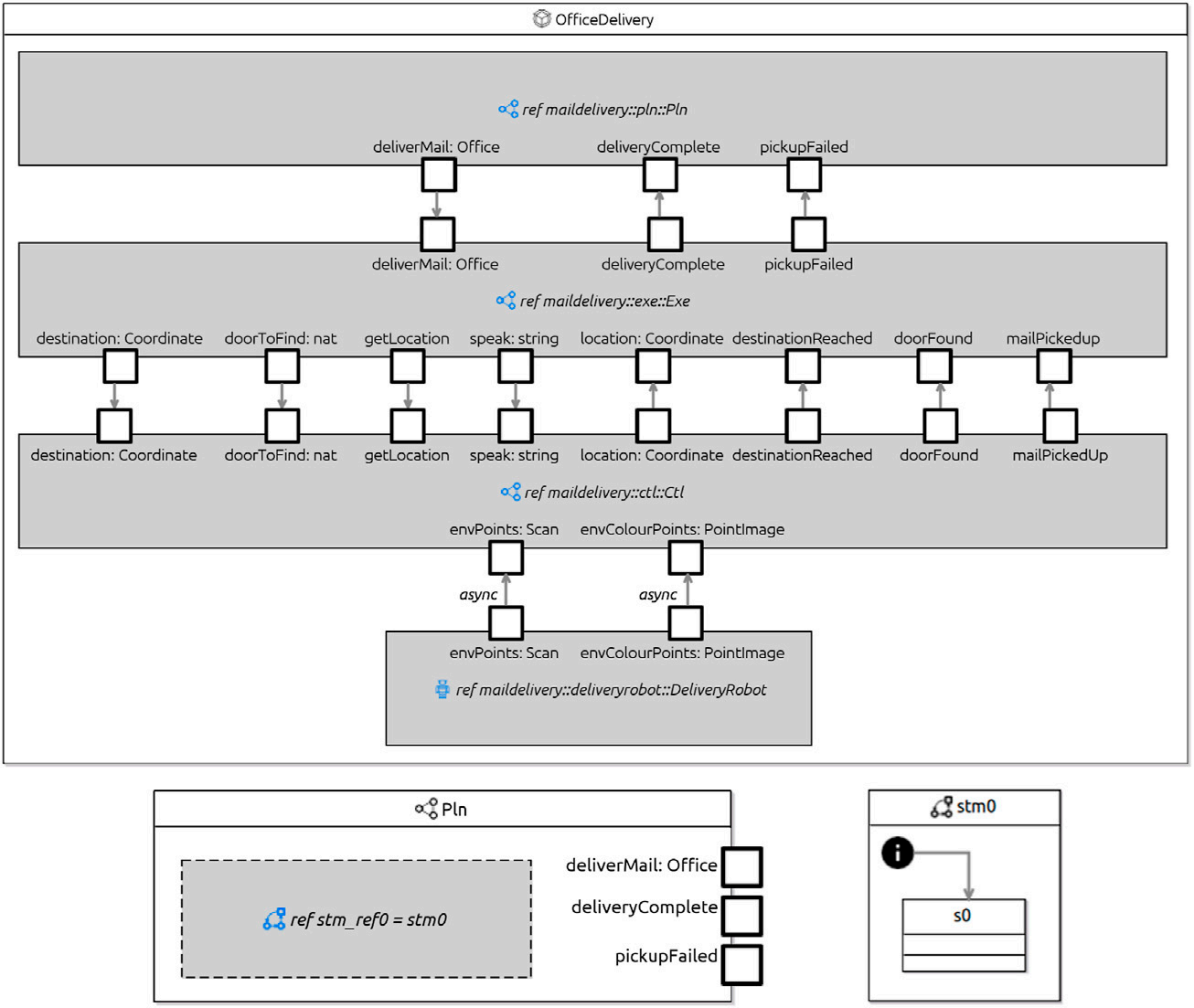
Delivery robot in RoboChart.

Table 3 presents an informal account of how RoboArch elements can be mapped to a RoboChart model. Transformation rules formalise this mapping, defining the (formal) semantics of RoboArch; their implementation allows the automatic generation of RoboChart models. RoboChart’s formal semantics underpins RoboArch and allows properties of a RoboArch design to be verified (see Figure 1). Figure 4 presents parts of the RoboChart model for the design in Listing 1.

**TABLE 3:** Mapping RoboArch to RoboChart.

RoboArch	RoboChart
System	Module, TypeDecl, Function, Interface, RoboticPlatform
Layer	Controller
inputs and outputs	Events
Connection	Connection

**FIGURE 5:**
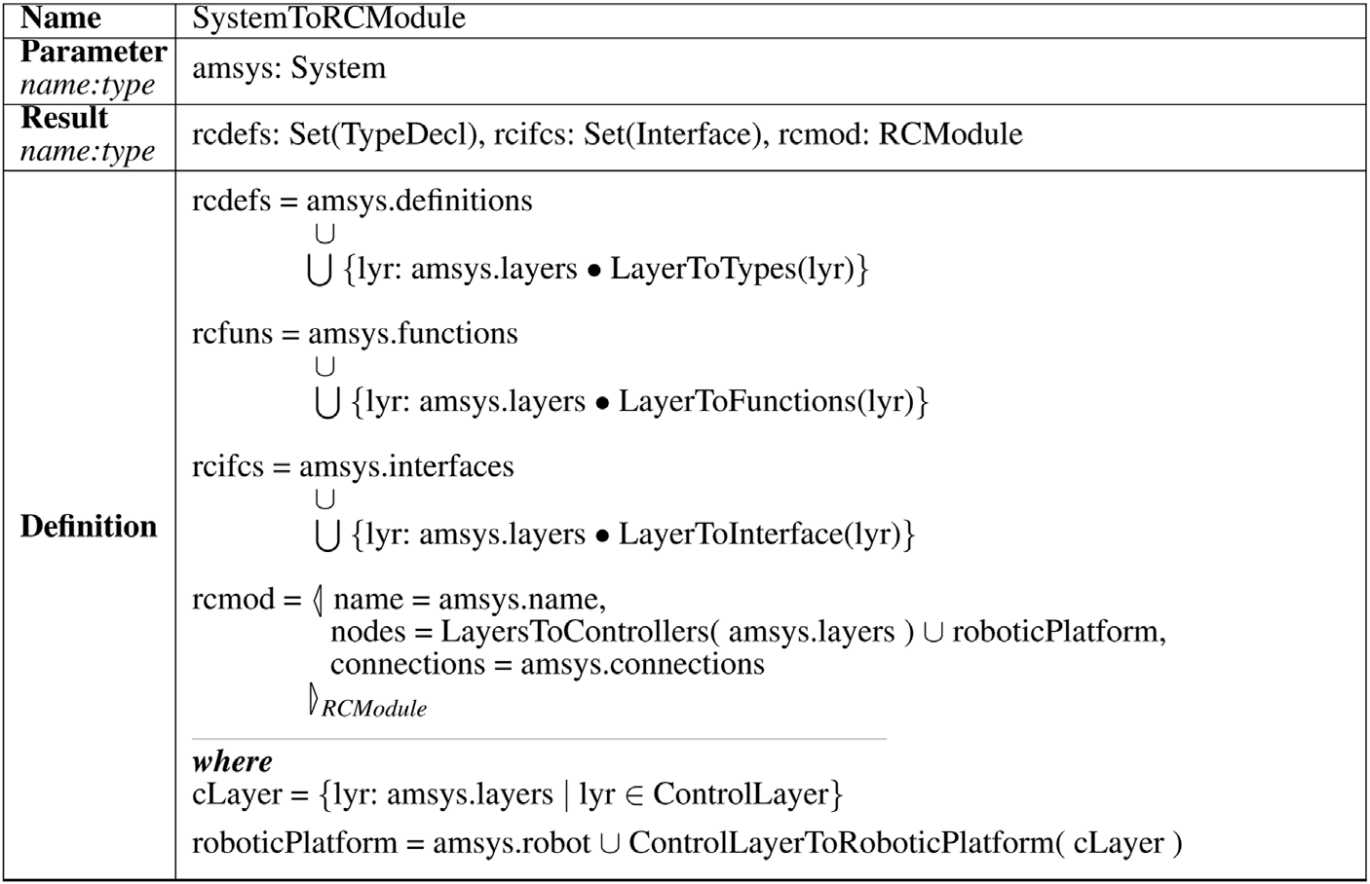
Example transformation rule.

The top-level transformation rule, shown in Figure 5, maps a RoboArch System to the RoboChart type definitions, functions, interfaces, and robotic platform that it declares. Importantly, the top rule defines a valid RoboChart module for the system. The mapping provides a graphical representation as well as a semantics for these elements, since RoboChart is a diagrammatic language. Due to space restrictions, we cannot present all the transformation rules, but they are available^4^.

A module is the RoboChart element representing a (parallel) robotic control software. In Figure 4, the module OfficeDelivery defines the RoboArch system of the same name.

A RoboChart module has its platform-independent behaviour characterised by a RoboticPlatform and one or more parallel Controllers whose behaviours are defined by one or more state machines running in parallel. The module defined by a RoboArch system includes a reference to its RoboticPlatform, and one Controller for each Layer. In Figure 4, the module has *ref*erences to the platform DeliveryRobot, and to three controllers Pln, Exe, and Ctl named after the layers of the RoboArch system.

The inputs and outputs of a Layer become events of the corresponding RoboChart controller. Connections between layers and the robot become connections between controllers and the robotic platform.

Figure 4 shows the RoboChart controller for the planning layer Pln. The events appear along the border of the controller. Inside, there is a reference to a single minimal machine stm0 because in this example no pattern is specified by the RoboArch design. The minimal machine, also shown in Figure 4, is a placeholder to be changed by the designer to specify their required behaviour. The minimal machine consists of a single initial junction, a state s0, and a transition that leads from the initial junction to s0.

For illustration, we show the top rule SystemToRCModule in Figure 5; it uses further rules (omitted here) to specify the RoboChart resulting elements rcdefs, rcfuns, rcifs, and rcmod that give the semantics of the system amsys given as input. The other rules are specified in the same style.

The resulting RoboChart type definitions rcdefs are the union of RoboArch system type definitions amsys. definitions and the generalised union (⋃) of the definitions resulting from applying a rule LayerToTypes to each RoboArch layer (amsys.layers). The types used in the rule definitions (TypeDecl, Interface, RCModule, and all others) are part of the RoboArch and RoboChart metamodels. They define the valid attributes (amsys.definitions, amssys layers, and so on). The definitions of the results rcfuns and rcifcs are similar to that of rcdefs, but use the rules LayerToFunctions and LayersToInterfaces.

The resulting RoboChart module rcmod is given by an object (specified by the construct ◁ _ ▷_*RCModule*_) whose attributes define the name, nodes (controllers and robotic platform), and connections. The name of the module is the system name amsys. name. The nodes are the controllers defined by applying the rule LayersToControllers to the system’s layers and a roboticPlatform as defined in the where clause. The connections of the module are those defined directly by amsys. connections.

The where clause defines the roboticPlatform to be the union of the RoboArch platform amsys. robot with the result of applying a rule ControlLayerToRoboticPlatform to the control layer cLayer. The platform amsys. robot is directly mapped to the RoboChart model. The layer cLayer is defined (*via* a set comprehension) as the layer lyr of amssys layers whose type is ControlLayer. The well-formedness conditions ensure that there is at most one such layer. With the use of ControlLayerToRoboticPlatform, we cater for the possibility that a pattern in the control layer extends the definition of the platform.

Although the translation of a layered design from RoboArch to RoboChart is reasonably direct, use of RoboArch, instead of constructing a RoboChart model from the start, has several advantages. RoboArch provides clear guidance on how to define and connect a robotic platform and the controllers; validation ensures definition of proper layers. On the other hand, translation to RoboChart provides support for verification. For example, we can prove that the RoboArch design is deadlock free.

In the next section, we show how we can enrich the definition of a layer.

## 4 Results: Reactive skills in RoboArch

With the RoboArch framework defined in the previous section, we can now formalise and use specific architectural patterns. In this section, we explain how to achieve that using the reactive-skill pattern for illustration. We first provide an overview of the pattern (Section 4.1), and then formalise it *via* a metamodel and well-formedness conditions (Section 4.2), and *via* transformation to RoboChart (Section 4.3).

### 4.1 Overview

The reactive-skills pattern can be used in the control layer, typically of a three-layer architecture (Bonasso et al., 1997). It combines deliberation and reactivity to improve robustness. The pattern has been used in a variety of applications: a robot to identify people and approach them (Wong et al., 1995), a trash collecting robot (Firby et al., 1995b), a robot that navigates a building (Firby et al., 1995a), and in the automation of remote-manipulation system procedures for the space shuttle (Bonasso et al., 1998). A framework that allows skills to be implemented using C, C++, Pascal, LISP and REX is available (Yu et al., 1994).

We characterise the reactive skills pattern by two concepts: skills and a skills manager. A *skill* performs an operation using input values, which can be from sensors or outputs of other skills. The skill’s output values can establish associations to and from the robotic platform, or be the result of applying a computational transform to the skill’s inputs. A set of skills is used together to accomplish a task identified in the dependant (typically executive) layer. A skills manager is a cyclic mechanism that coordinates communication between skills and provides an interface for the dependant layer to: run the skills required for a task, receive notifications from monitored events, and set and get parameter values of skills.

Skills can be of one of two types: D-Skill or C-Skill (Yu et al., 1994). D-Skills interface physical devices such as sensors and actuators with the other skills of the control layer; their input values are actuation commands and their output values are sensor data. C-Skills execute a computational transform using the skill’s inputs to determine its outputs. By the monitoring of skills, the manager triggers events on desired conditions becoming true. Table 4 describes elements used by skills and skills managers.

**TABLE 4:** The elements of reactive skills.

Element	Description
Initialisation routine	When the system starts, the skill initialises itself
Startup	A skill performs required startup procedures each time it is activated
Reply	Response from the control layer to the dependant (executive) layer
Cleanup	When a skill is disabled, cleanup actions are performed
Parameter	A variable that allows a skill’s behaviour to be adjusted by the dependant layer
Input	Receives the value of a data type
Output	A resulting value that contributes to the robot’s behaviour
Computational transform	Once activated the skill continually computes its outputs from its inputs
Activate function	Allows a skill to be activated
Deactivate function	Allows a skill to be deactivated when it is no longer required

In Listing 2, we sketch the design of the control layer of MailDelivery using reactive skills to specify the behaviours regarding moving the delivery robot to a given target location. The type of pattern specified by the pattern clause determines the subsequent clauses that can be used. For reactive skills, the subsequent clauses are skills, connections, and monitors.

Every skill has a unique name, and optionally parameters, a priority, inputs, and outputs. The skills clause declares the skills. There are separate clauses for defining each type of skill: dskill for D-Skills and cskill for C-Skills. In Listing 2, we show three D-Skills named Move, ColourVision, and Proximity, and one C-Skill DetermineLocation.

RoboArch dskills and cskills declare inputs and outputs using the inputs and outputs clauses. A skill communicating a value to a dskill’s input results in the physical state of the device that the dskill represents being potentially affected. In our example, a value communicated to the Move D-Skill velocity input results in the velocities of the motors in the robot’s base being set.

A value from a dskill’s output represents the state of the environment, as sensed by the device the dskill represents. In our example, a value received from the Proximity skill’s envPoints output determines a range of distances to surfaces in the delivery robot’s field of view.

A C-Skill uses its inputs to compute its outputs resulting in behavior that can be used to accomplish parts of a task. In our example, the DetermineLocation skill takes a colour image of the environment and using an image-based localisation technique calculates the coordinates of the delivery robot. To perform this function, DetermineLocation has one input image of type PointImage, and one output location of type Coordinate. The computational transform that specifies the behaviour of C-Skills can be defined by customising the generated RoboChart model.

Listing 2 Reactive skills movement.

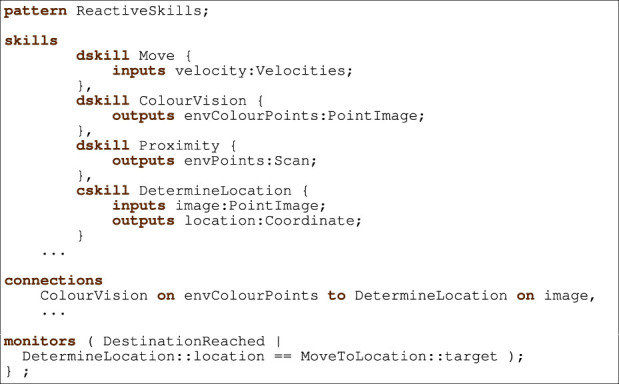

Skills can communicate with each other *via* the skills manager. The source and destination of the communication (skills’ inputs and outputs) are determined in a connections clause. Each connection is unidirectional and relates an input of one skill to the output of another. Our example declares a connection from the ColourVision output envColourPoints to the DetermineLocation’s image input.

Layers that depend on a reactive-skills control layer may need to monitor for particular conditions becoming true. To minimise the frequency at which the dependant layer needs to check the conditions, the reactive-skills pattern provides events that are independently triggered to notify the dependant layer of the occurrence of any monitored conditions. The monitors clause declares the monitors for the layer. They have a name and specify the logical condition to be monitored in terms of skill outputs and parameters. For our example, a condition that is monitored is the arrival of the delivery robot at the target location. A monitor DestinationReached has a condition that evaluates to true when the location output of the DetermineLocation skill is equal to the target parameter of a MoveToLocation skill.

Next we describe the RoboArch metamodel and well-formedness conditions for reactive-skills designs.

### 4.2 Metamodel and well-formedness conditions

**FIGURE 6:**
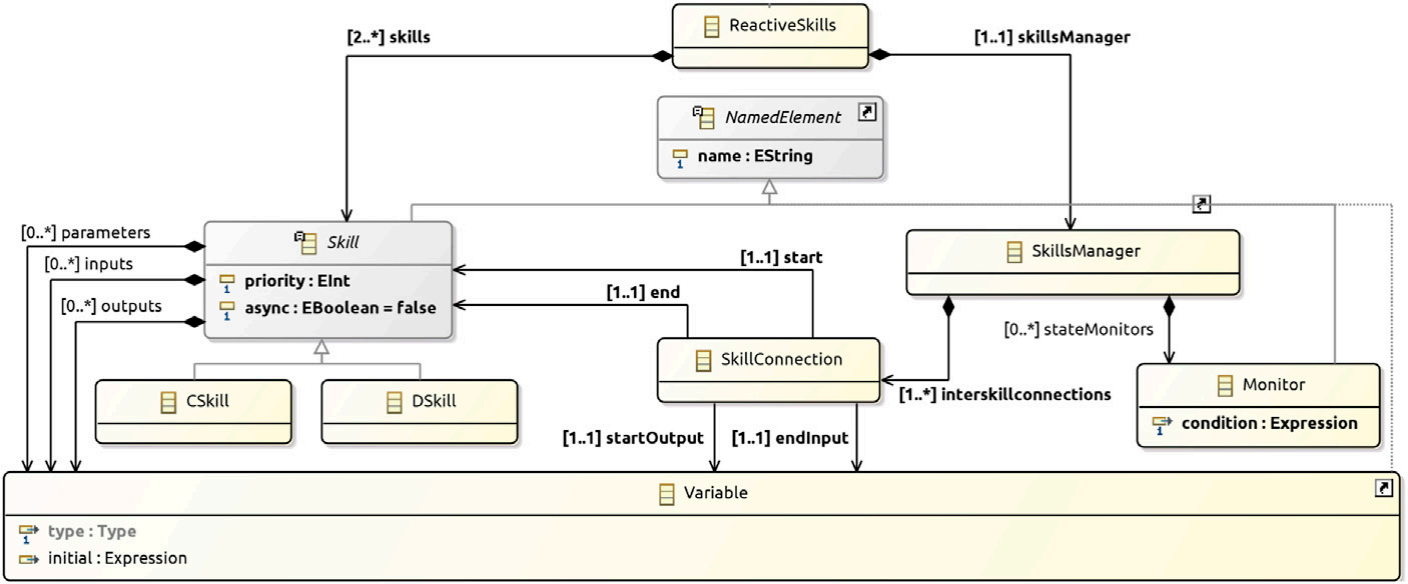
Reactive skills metamodel.

The class ReactiveSkills representing a reactive-skills design is a subclass of Pattern (see Figure 3). Figure 6 details its definition, giving a precise characterisation of the pattern.

In a ReactiveSkills design there must be at least two skills and exactly one skillsManager. Skills can have parameters, inputs, and outputs all represented by Variables. Skills can be asynchronous and have a priority.

Skill is an abstract class (it has no objects). Its subclasses CSkill and DSkill are the concrete classes, whose objects are subject to different well-formedness conditions presented below.

The SkillsManager establishes one or more interskillconnections and may have stateMonitors. Monitors have a name and a condition defined by a RoboChart expression. SkillConnections associate Skills defining the start and end of the connections, the output startOutput of start, and the input endInput of end.

The well-formedness conditions that apply to reactive-skills designs are presented in Table 5. RS1 and RS2 ensure the use of reactive skills as intended to provide the essential behaviours that use the sensors and actuators (*via* the services of robotic platform), which other layers depend on to carry out the robot’s tasks. RS3 records that the inputs of the D-Skills correspond to events of the robotic platform. RS4 is needed because a C-Skill or D-Skill in isolation can perform no meaningful function that alters the state of the robot or its environment. A C-Skill requires a D-Skill in order to interact with a sensor or actuator *via* the services of the robotic platform. With RS5 and RS6, we ensure that every skill contributes to the behaviour of the system. RS7 to RS10 ensure that connections are between inputs and outputs of different skills of the right type. Finally, RS11 ensures that monitors are concerned with skill data.

**TABLE 5:** The well-formedness conditions of reactive-skills designs.

Condition	Description
**RS1**	A Layer that has a pattern of type ReactiveSkills must be a GenericLayer or ControlLayer
**RS2**	For a Layer with pattern type ReactiveSkills, at least one of the System’s connections is from that layer to a RoboticPlatform or that layer has at least one rinterface
**RS3**	For each event of ReactiveSkills Layer’s interfaces, there must be a DSkill input with a matching name
**RS4**	ReactiveSkills must contain a CSkill and a DSkill
**RS5**	A CSkill must have at least one output
**RS6**	A DSkill must have at least one output *or* input
**RS7**	The start and end Skill of a SkillConnection must be distinct
**RS8**	The startOutput of a SkillConnection must be an output of its start Skill
**RS9**	The endInput of a SkillConnection must be an input of its end Skill
**RS10**	The types of the startOutput and endInput of a SkillConnection must match
**RS11**	A Monitor’s condition must only refer to parameters, inputs, and outputs of the Skills

Valid reactive-skill designs, that is, those that satisfy the above well-formedness conditions, can be transformed to (and so formally described as) a RoboChart model, as described in the next section.

### 4.3 Reactive skills in RoboChart

Rules that can be used to transform a reactive-skill design to RoboChart are available^5^. Here we give an overview of our approach formalised by the rules in modelling reactive-skill designs in RoboChart.

A RoboChart controller representing a layer that uses the reactive-skills pattern has one state machine for the skills manager, and one machine for each skill. The skills-manager machine has events to manage the activation and deactivation of skills, receive parameter values, and communicate monitor-event and information replies. A skill machine has events for each of its inputs, outputs, and parameters.

**FIGURE 7:**
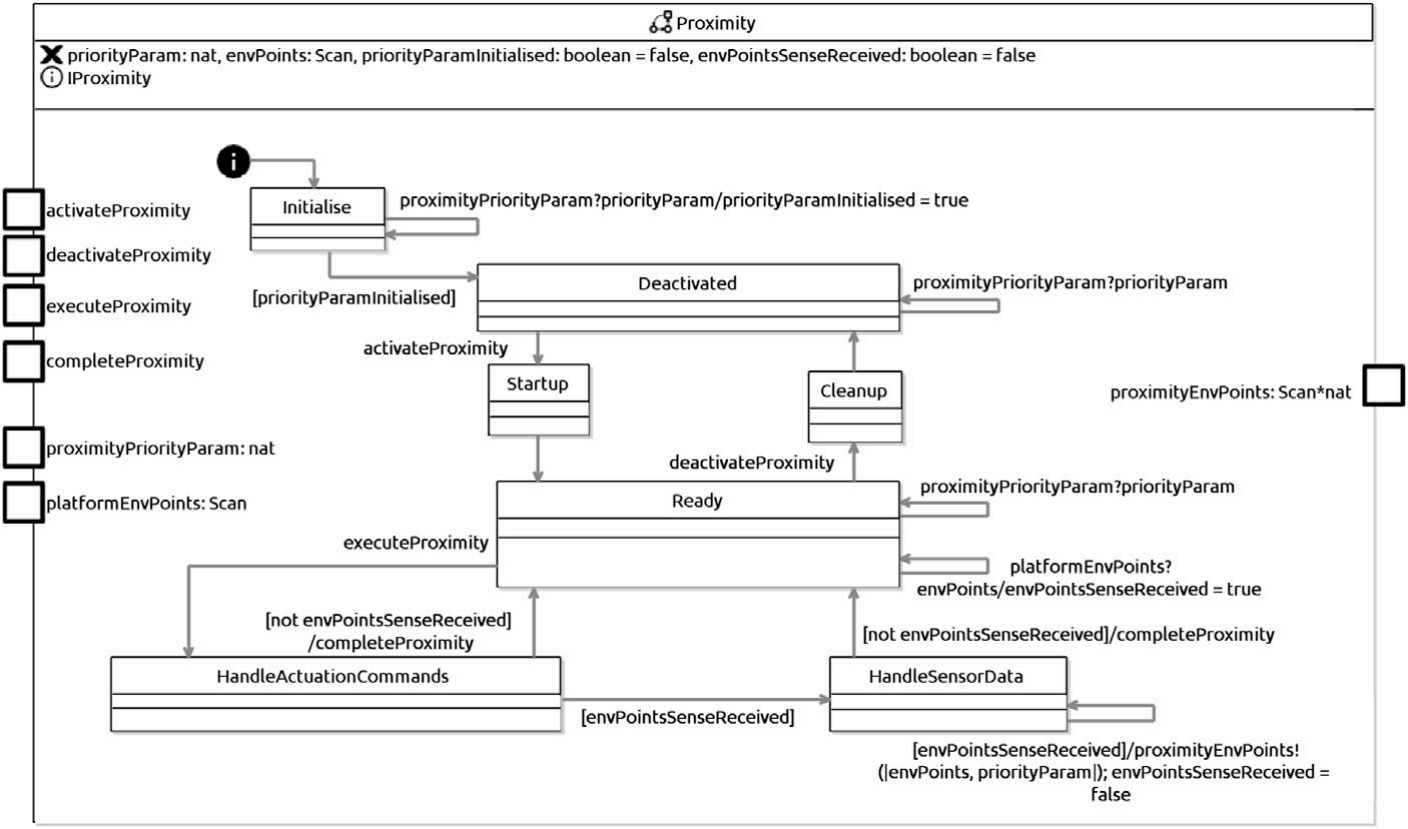
RoboChart state machine for Proximity D-Skill.

As an example, Figure 7 shows the machine for Proximity in Listing 2. That machine reflects the description of the design pattern summarised in Table 4, and is representative of the state machines that are automatically generated for D-Skills. The declaration at the top in Figure 7 introduces variables priorityParam, to record an input priority value, envPoints, to record the output of the skill, and booleans priorityParaminitialised and envPointsSenseReceived, recording information about inputs. An interface IProximity declares the events used to exchange information with the skills-manager machine.

A D-Skill state machine starts at the state Initialise, where it accepts a priority for the skill *via* an event: in our example, proximityPriorityParam. When that input is taken, the variable priorityParaminitialised is updated to record that. Once that variable has value true, a transition to the state Deactivated becomes enabled, and is taken. In Deactivated, the priority can still be updated, until the skills manager raises an activate event (activateProximity in the example), when the machine moves to the state Startup.

Typically, the designer needs to enrich the state Startup to add the actions that the skill carries out at start up, perhaps *via* an entry action, or *via* a state machine making Startup a composite state. When those actions complete their execution, a D-Skill state machine moves to the state Ready.

In Ready, a new priority and inputs from the platform may be received. In the example, the value envPoints may be received from the platform *via* an event platformEnvPoints. When that happens, the value of a corresponding Boolean variable, here envPointsSenseReceived, is updated. This can go on until the skills manager raises an execute event (executeProximity, in the example) when the machine moves to the state HandleActuationCommands. Actions there, defined by the designer, might deal with buffering, for example. If, however, no input has been received (just not envPointsSenseReceived in Proximity), the machine flags that the skill has completed its task (completeProximity) and goes back to Ready.

If an input has been received, the machine moves to HandleSensorData. In general, HandleSensorData may deal with several pieces of data coming from the platform. All those that have been received may be communicated to another skill, together with its priority. In our example, we have just envPoints, which is output *via* proximityEnvPoints. When that happens, the value of envPointsSenseReceived is updated back to false. When all data has been communicated, a D-Skill machine moves back to Ready.

Variations of the D-Skill state-machine definition take into account D-Skills that can output to the platform, and also D-Skills that have several inputs or several outputs.

**FIGURE 8:**
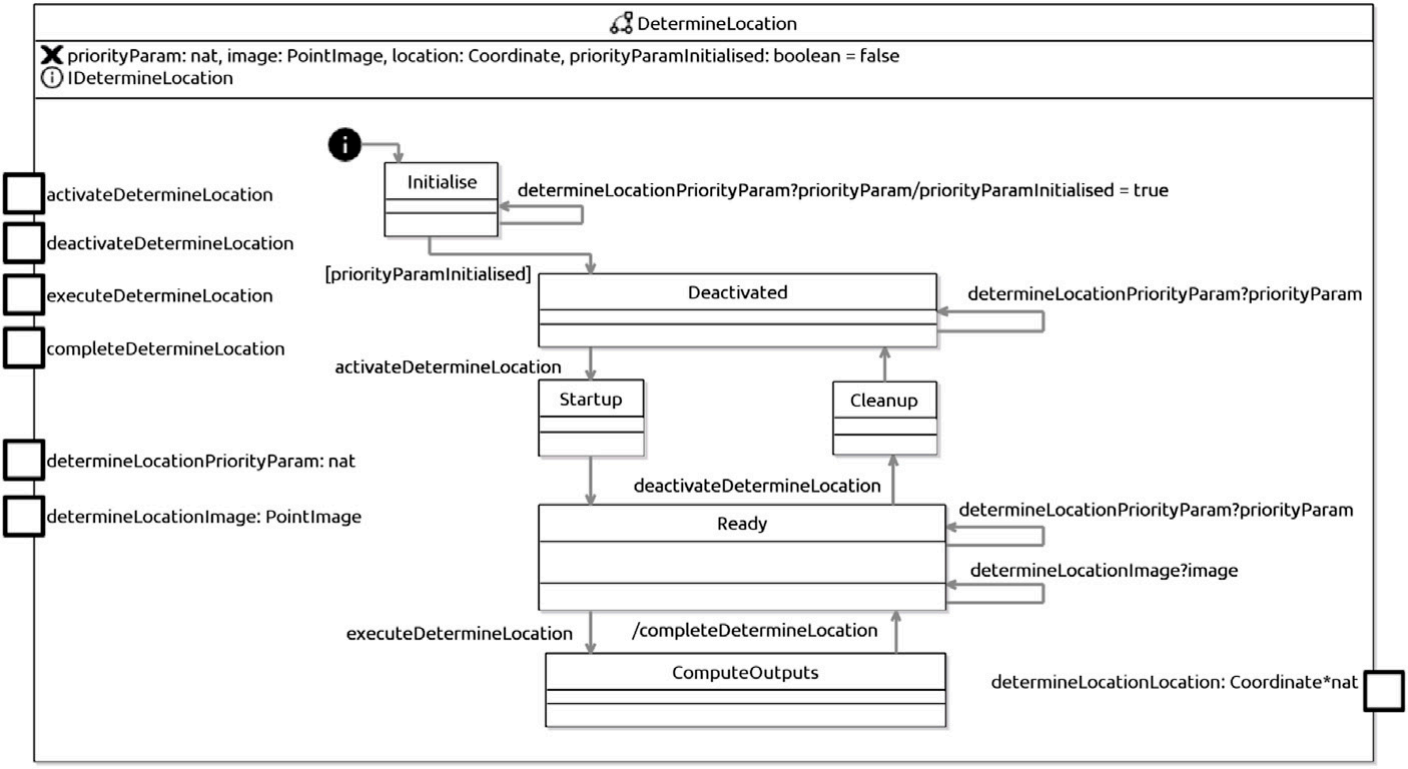
RoboChart state machine for DetermineLocation C-Skill.

A machine for a C-skill is shown in Figure 8. It is very similar to that of a D-Skill; the difference is that, instead of states HandleActuationCommands and HandleSensorData to deal with inputs and outputs of the platform, we have a single state ComputeOutputs. When the skills manager raises the execute event (executeDetermineLocation in the example), the machine moves to ComputeOutputs.

The designer must complete the definition of this state to reflect the calculations to be carried out by the skill. Once they finish executing, the machine returns to the state Ready, having signalled completion to the skills manager *via* a complete event: completeDetermineLocation in the example.

**FIGURE 9:**
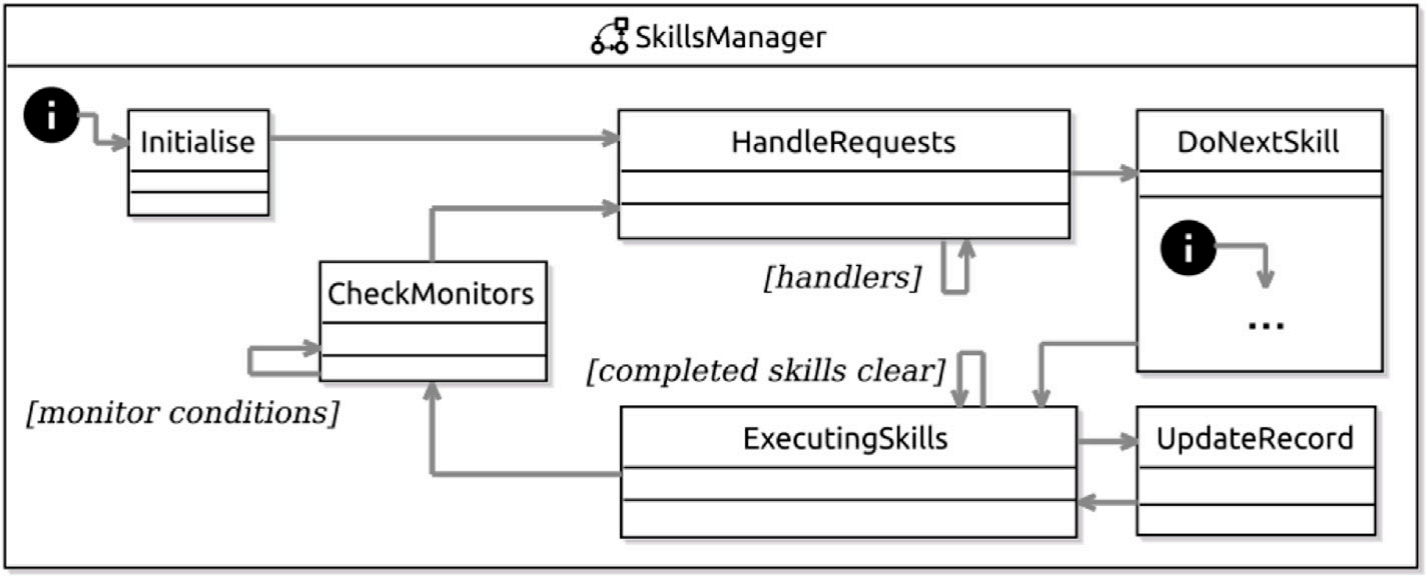
RoboChart skills-manager machine for the example.

Finally, Figure 9 sketches the machine for the skills manager. The complete machine for our example that can be automatically generated is too large to include here. In the sketch, we show that a skills-manager machine starts in the state Initialise, in which it sets local variables, such as cycleSkills, recording the skills to execute in the next cycle. Afterwards, the skills-manager machine moves to HandleRequests.

In the state HandleRequests, for each request, there is a transition triggered by an event that represents a request from the dependant layer, whose transition action provides the required information, or updates variables to record the request: activate or deactivate skills, initiate event monitor, stop event monitor, or set skill parameters. Once the amount of time defined by the cycle of the skill manager is past, the machine moves to DoNextSkill. The cycle time is defined by a constant, whose value can be defined by the designer or left unspecified (until simulation or code generation).

DoNextSkill is a composite state that uses the cycleSkills variable to start all skills that are to execute in the current cycle. In the DoNextSkill machine, there is a state for each skill that sends its input values, raises the event that starts its execution (such as executeDetermineLocation) and updates a variable executingSkills. When all skills are set, cycleSkills gets empty, and the transition to ExecutingSkills is taken.

The state ExecutingSkills accepts the outputs of skills while they are executing. When an output is received, the machine moves to the state UpdateRecord, where the inputs to which the received output is connected are updated. This is done using an UpdateValue function (omitted here) that only updates the input if it is the first update of the cycle or if the new value comes from a skill with higher priority. After each update, the machine moves back to ExecutingSkills.

The state ExecutingSkills also accepts completion events from the skills (such as completeDetermineLocation), updating the executingSkills variable after each such event. When all skills have completed execution, executingSkills is empty and the transition to CheckMonitors is taken.

In CheckMonitors, there is a transition for each monitor. If a monitor condition occurs, a corresponding event notifies the depending layer. When all monitors are checked, the machine moves back to the state HandleRequests, after reinitialising variables such as cycleSkills.

Using the semantics of RoboChart that is automatically generated, we can prove properties of the design. We have, for example, proved deadlock and livelock freedom, and some other trace-based properties of some of the machines that are automatically generated. In these proofs, we can cater for general properties of any design, and for application-specific properties.

In the next section, we discuss how we can define and formalise a CorteX-based design pattern in RoboArch, opening the same possibilities for CorteX and CorteX designs (see Figure 1).

## 5 Discussion: CorteX and RoboArch

As already said, CorteX is a framework tailored to the development of complex nuclear robotic systems. It primarily focuses on data representation and communication to solve issues of maintainability and extensibility. In this section, we discuss the integration of CorteX and RoboArch.

We envisage two main approaches for integrating RoboArch and CorteX. The first supports the generation of CorteX implementations of RoboArch models (Section 5.1). The second approach extends RoboArch to support modelling CorteX architectures (Section 5.2).

### 5.1 From RoboArch to CorteX

As discussed in Sections 3, 4, the semantics of RoboArch is specified in terms of RoboChart, which opens the possibility for the generation of several artefacts (see Figure 1). We can obtain automatically mathematical models for verification, such as CSP (Miyazawa et al., 2019) scripts, for verification of reactive and timed properties, and PRISM (Ye et al., 2021) reactive modules, for verification of probabilistic properties. We can also obtain code (Li et al., 2018) and RoboSim models describing simulations (Cavalcanti et al., 2019). RoboSim is a sister notation of RoboChart tailored to the design and verification of simulations with a similar component model and artefact-generation facilities.

A code generator that produces CorteX-compatible implementations of a RoboArch model can take advantage of some of the abovementioned functionalities. The first step requires the generation of the semantics of the RoboArch model in RoboChart as described in this paper. Since CorteX is a cyclic architecture, it is useful to transform (automatically) the resulting RoboChart model into a simulation model, written in RoboSim, *via* the RoboStar correctness-preserving model-to-model transformation. Next, we can use one of the RoboSim model-to-model transformations to generate an intermediate representation of imperative code and a model-to-text transformation tailored for CorteX. Currently, two transformations targeting the programming languages C and Rust are under development.

With the use of the intermediate representation, we guarantee that the semantics of RoboChart and RoboSim is preserved by the code. This follows from the fact that the generation of the intermediate representation is a mechanisation of the RoboSim semantics, and the model-to-text transformation is direct. For CorteX, each state machine can be implemented as a simplex, the basic unit of data and behaviour in CorteX code. This approach matches well the parallel paradigms of RoboChart and CorteX.

On the other hand, the translations from RoboChart to RoboSim and from RoboSim to the intermediary representation give rise to additional parallel components for orchestration of operation calls and during actions inside state machines. This can create an overhead in the target code. If this overhead becomes an issue, we can alternatively, directly convert the RoboSim model into code *via* a generator specifically tailored for CorteX. While this alternative involves significantly more work (as it does not reuse the existing intermediate representation generator), it allows for more control over the structure of the CorteX implementation, and a one-to-one match between state machines and simplexes.

In the approaches above, CorteX is used as a target middleware. An alternative explored in the next section is the use of CorteX concepts already at the design level, giving rise to an architectural pattern for CorteX. This enables design and verification for CorteX.

### 5.2 CorteX in RoboArch

A CorteX implementation does not explicitly have the notion of layers. In fact, one might even argue that a layered architecture is incompatible with CorteX due to its distributed nature. This, however, is not the case, since layers are not necessarily centralised or co-located, and a layer or set of layers can be deployed as a distributed system. Moreover, well-designed code separates planning and control functionality. It is, therefore, beneficial to use separate sets of CorteX simplexes to deal with planning and control.

For this reason, the use of layers does not prevent the adoption of CorteX, and, moreover, embedding CorteX designs in RoboArch as a pattern for any layer provides extra support to address the interoperability issue with non-CorteX applications such as ROS (Caliskanelli et al., 2021, *p*. 320). The use of a layered RoboArch design can help to ensure not only that code for planning and control is kept separate, but that a strict layered discipline is enforced, even if the code, as it is often the case, does not have a notion of layer.

**FIGURE 10:**
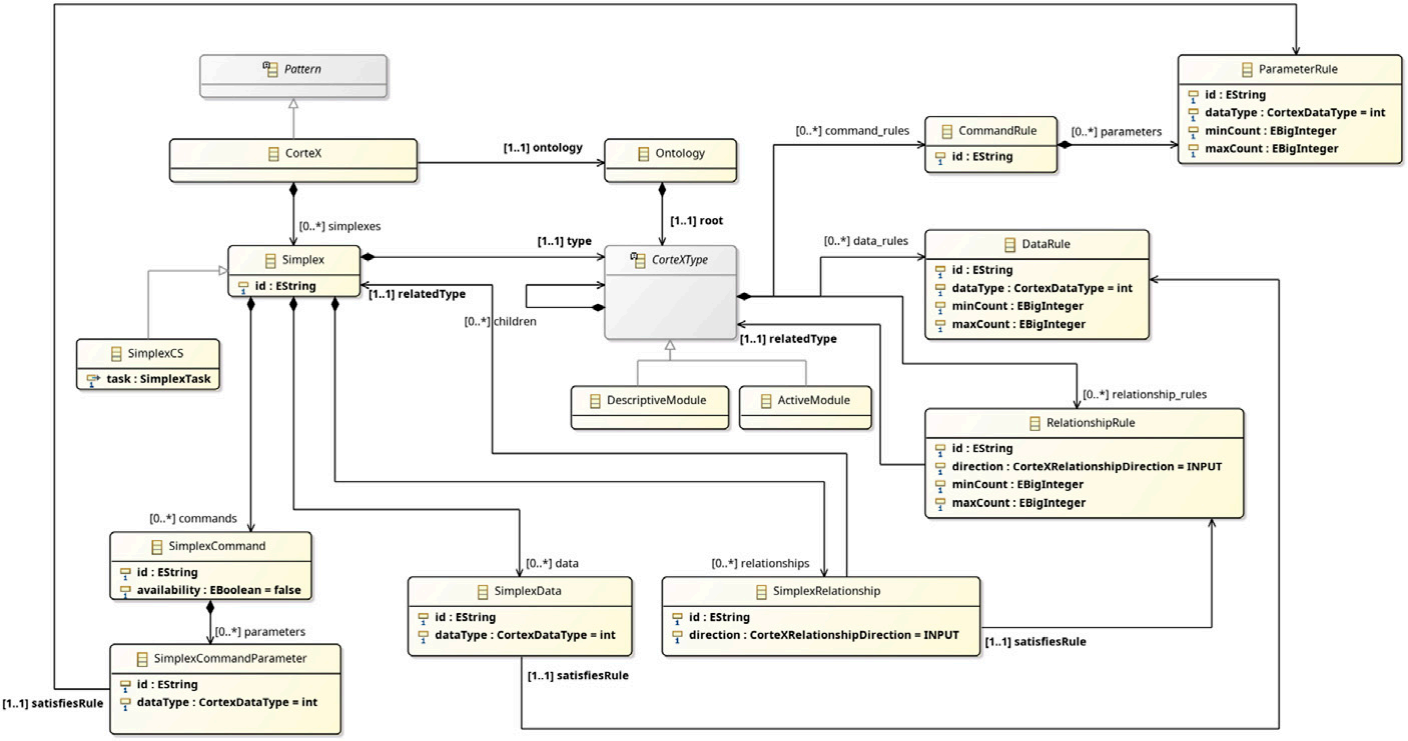
Metamodel of CorteX for integration with RoboArch.

Figure 10 depicts a metamodel for integrating CorteX into RoboArch; it is based on the description of CorteX in (Caliskanelli et al., 2021). As for reactive skills, we model the CorteX architecture as a RoboArch Pattern. Listing 3 and Listing 4 present the sketch of a layer CorTeXl that uses the CorteX pattern in the design of a simple application based on mobile robots inspired by an example in (Caliskanelli et al., 2021).

Listing 3 Mobile robots ontology.

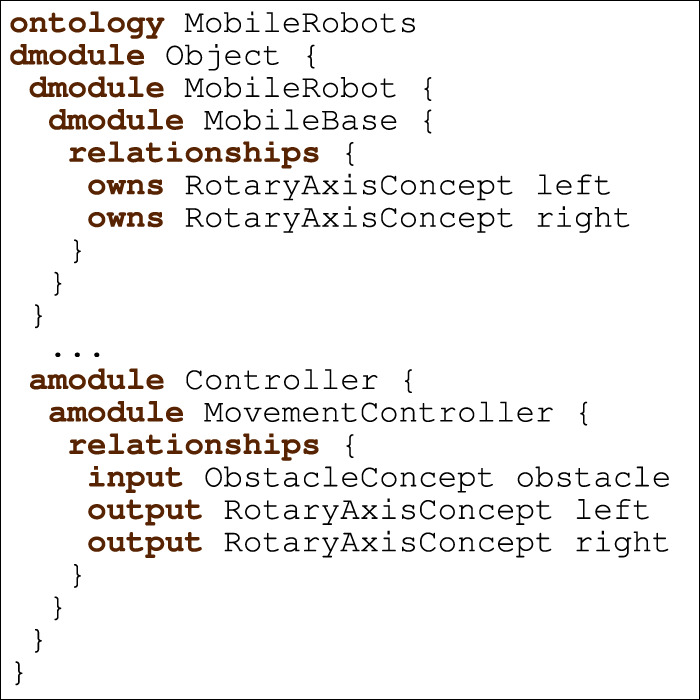

Listing 4 Example of layer using the CorteX pattern with MobileRobots ontology.

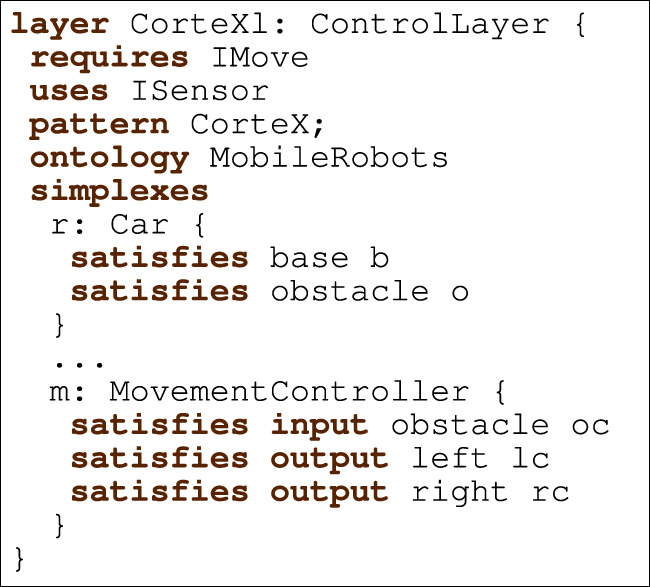

As discussed in Caliskanelli et al. (2021, pp. 317–319), a CorteX application is parameterised by an ontology, represented here by the attribute ontology. An object of class Ontology has a single attribute root of type CorteXType, which is an abstract class that can be realised as either a DescriptiveModule or an ActiveModule. The distinction is similar to that between passive and active classes.

A CorteXType may contain any number of CortexType children and sets of rules applicable to commands, data, and relationships. An ontology is, therefore, structured as a tree, a hierarchy of concepts akin to an object-oriented model. The rules establish constraints over CorteXTypes. A CommandRule has an identifier (for instance, move) and some parameters. Each ParameterRule defining a parameter has itself an identifier and a dataType (limited to integer, float, Boolean, or string), possibly an array, as defined by the attributes minCount and maxCount. We omit here the simple enumeration CortexDataType.

A DataRule is similar to a ParameterRule. Finally, a RelationshipRule describes a relationship with a CorteXType, defined by relatedType. It specifies a direction, using a value of an enumeration type CorteXRelationshipDirection including INPUT and OUTPUT, and a multiplicity.

Listing 3 shows an excerpt of the ontology for the example. It includes descriptive modules, such as MobileBase, and active modules, such as MovementController. MobileBase represents a two-wheeled robot and contains two concepts of type RotaryAxisConcept, which represent the data associated with the left and right wheels. The module MovementController specifies an input concept of type ObstacleConcept and two output concepts of type RotaryAxisConcept (both of these concepts are specified in the ontology as descriptive modules, but omitted in Listing 3).

A CorteX pattern also contains a set of simplexes. A Simplex has an identifier, a type from the ontology, and sets of data, relationships, and commands. A SimplexData models a piece of primitive data containing an identifier and a dataType (limited to integer, float, Boolean, string, and possibly an array). The attribute satisfiesRule identifies a DataRule of the ontology that is implemented (satisfied) by the simplex.

A SimplexCommand has an identifier, availability, and a set of parameters. Like for SimplexData and DataRule, a SimplexCommandParameter satisfies a ParameterRule.

A SimplexRelationship describes a connection between two simplexes, namely, the simplex that contains it and the simplex identified by relatedType. In addition, a SimplexRelationship satisfies a RelationshipRule.

Listing 4 depicts the RoboArch control layer CorTeXl that uses the CorteX pattern and refers to the MobileRobots ontology in Listing 3. It requires interfaces IMove and ISensor; the first declares operations setLeftMotorSpeed and setRightMotorSpeed, and the second the event obstacle. Next, CorTeXl specifies its pattern (CorteX) and the pattern’s components. These are the ontology (MobileRobots) and the set of simplexes. Each simplex has a name and a type from the ontology, and information about how the ontology relationship rules are satisfied. For instance, MovementController has name m and specifies three relationships; the first specifies that the relationship rule left (of the module MovementController in Listing 3) is satisfied by the simplex lc (declared in the pattern but omitted in Listing 4) of type RotaryAxisConcept.

A SimplexCS is a Simplex with a notion of task, which defines behaviours to be executed in particular points of its lifecycle. This is similar to D-Skills in the reactive-skills pattern, where the top-level execution protocol of the D-Skill is fixed and user-defined behaviours are run in particular stages of this protocol.

In our CorteX metamodel, we omit the concept of a ClusterCS, which is related to allocation of simplexes to computational units. This is an issue not covered in RoboStar technology. Automatic generation of CorteX code may, for example, define a simple default allocation of simplexes to a single computational unit for further elaboration by the CorteX designer later.

Additionally, we omit the notion of Simplex Trees. These are sets of simplexes, which are represented in our metamodel by the attribute simplexes of CorteX. So, each layer that uses a CorteX pattern has a single set of simplexes. With this metamodel, different sets can, and need to be, allocated in different layers. Further experience may indicate that we need several sets of simplexes in a layer, if the layer discipline turns out to be too restrictive in some cases. This simple extension is left as future work.

There are three well-formedness conditions that apply to a CorteX design as defined below. They are all related to the data, commands, and relationships of a Simplex and the rules that they indicate that are satisfied by them. Together the conditions ensure that the rules used in a Simplex are well defined.

**C1** The DataRule of a SimplexData is in the CorteXType of its Simplex.

**C2** The ParameterRule of a SimplexCommandParameter is in the CorteXType of its Simplex.

**C3** The RelationshipRule of a SimplexRelationship is in the CorteXType of its Simplex. In addition, the Simplex defined by its relatedType has the type defined by the relatedType of the RelationshipRule.

For designs that satisfy these restrictions, we can define a RoboChart sketch *via* transformation rules. The semantics of the CorteX pattern would be specified in RoboChart in line with the semantics of RoboArch. Each descriptive Simplex, that is, a simplex whose type is a DescriptiveModule, is specified by a RoboChart data type, and each active Simplex (ActiveModule type) is defined by a state machine, where the SimplexCommands are modelled as events, the SimplexData as variables, and the SimplexRelationships as connections. The semantics of a SimplexCS is specified by a state machine that enforces the specific execution protocol in a similar manner as done for D-Skills, which is illustrated in Figure 7.

As indicated in Figure 3, a layer contains input and output events for inter-layer communication. CorteX, on the other hand, does not use the same communication mechanism and requires a component to transform and route data between the layer and the CorteX application. This component can also be automatically generated similarly to how the semantics of RoboArch specifies the SkillsManager (Figure 9) for the reactive-skills architecture. Such a component partially solves the interoperability between different architectures; for instance, it allows the control layer in Figure 4, which uses the reactive-skills pattern, to communicate with an executive layer that uses a CorteX pattern.

To conclude, by allying RoboArch and CorteX, we can support the use of CorteX principles from an early stage of design. We can also support verification and automatic code generation. In this way, we further the CorteX agenda by supporting the development of traceable evidence of core properties of applications. Future work will consider significant case studies and automation.

## Data Availability

Publicly available additional rules were used in this study. They can be found at: https://robostar.cs.york.ac.uk/publications/reports/roboarch_rules.pdf.

## Footnotes

1
robostar.cs.york.ac.uk/robotool/

2 Laboratory for Analysis and Architecture of Systems CNRS.

3
https://robostar.cs.york.ac.uk/case_studies/

4
https://robostar.cs.york.ac.uk/publications/reports/roboarch_rules.pdf

5
https://robostar.cs.york.ac.uk/publications/reports/roboarch_rules.pdf
